# Isotretinoin for the treatment of facial lichen planopilaris: A new indication for an old drug, a case series study

**DOI:** 10.1002/ccr3.3210

**Published:** 2020-08-05

**Authors:** Farahnaz Fatemi, Fatemeh Mohaghegh, Farzaneh Danesh, Mina saber, Parvin Rajabi

**Affiliations:** ^1^ Department of Dermatology School of Medicine Isfahan University of Medical Sciences Isfahan Iran; ^2^ Pathology Department Faculty of Medicine Isfahan university of Medical Sciences Isfahan Iran

**Keywords:** frontal fibrosing alopecia, Lichen planopilaris

## Abstract

Despite the little information about the facial papules due to Lichen planopilaris (LPP), we have many cases with facial skin roughness in which histological study has showed LPP. Additionally, in those patients treating for frontal fibrosing alopecia or scalp LPP there was no improvement in facial papules.

## Graphical abstract

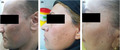

## 1 INTRODUCTION

Lichen planopilaris is an inflammatory skin disease in which involvement of hair follicles results in patchy scarring alopecia. Frontal fibrosing alopecia is a cicatricial alopecia affecting frontotemporal hairlines considered as a variant of LPP. Concomitant involvement of facial vellus hairs has been recently reported, presented as facial papules, which in some studies has been treated successfully with isotretinoin. It seems facial papules may response to oral isotretinoin. However, there are some reports of facial LPP in the absence of scalp LPP or frontal fibrosing alopecia (FFA). The primary objective of this study was to analyze the differences in clinical presentations of facial LPP. The secondary objective was to evaluate efficacy of oral isotretinoin in patients with facial, and tertiary objective was to report if we find any case of facial papule in the absence of Frontal fibrosing alopecia. This case series study was performed on 19 patients with facial papule referred to Alzahra hospital and clinics affiliated with Isfahan University of Medical Sciences in Isfahan‐Iran during 2018‐2019. The patients were treated with oral isotretinoin 20 mg for 6 months and clinical response of patients to treatment evaluated with a blinded dermatologist. Except for two males, all patient in this series were female (41.17% premenopausal and 58.82% postmenopausal) with mean age of 49.3 years. LPP involved the scalp in 29.4% and FFA observed in 52.9% of patients. There were 9 patients (47.36%) without any scalp involvement. Global Improvement Scale assessments at 6 months after baseline indicated that in all patients the lesions were significantly reduced after 6 months of treatment. According to the blinded dermatologist's opinion, 42.1% of patients had a good response and 26.3% had a very good response. Also, there were 9 patients with facial Lichen planopilaris without scalp involvement or frontal fibrosing alopecia (47.36%). Oral isotretinoin was an effective and safe treatment in patients with facial LPP. There were some patients with only facial involvement without FFA or scalp LPP.

Lichen planopilaris is the most common scaring alopecia characterized by lymphocytic infiltration around hair follicles. Although it is considered as an autoimmune disease, its exact pathogenesis mechanism is still unknown.^1^ Frontal fibrosing alopecia (FFA) is a distinctive form of primary lymphocytic cicatricial alopecia, which is considered as a variant of LPP.^2^ Moreover, FFA has markedly increased over the last years and is currently considered as “a growing epidemic” disease.^3^, ^4^ Accordingly, it mostly affects postmenopausal women, but it is also described among premenopausal women and men.^3^ Moreover, its association with autoimmune diseases has also been reported (eg, hypothyroidism).^5^, ^6^ Eventually, the number of the cases with FFA has increased in recent years as a result of its incidence rising.^7^ In addition, it has been considered as a variant of LPP that involves scalp hairs in frontotemporal hairlines, eyebrows, and eyelashes. The involvement of facial vellus hairs presented as skin‐colored follicular papules due to lichenoid perifollicular inflammation was described for the first time by Donati et al in 2011.^8^ Besides, it may be accompanied with body vellus hair involvement suggesting that FFA has more pathological expansion.^9^, ^10^, ^11^ Also, facial vellus hair involvement is reported as a clinical feature of FFA in the majority of studies; however, there are some rare reports of facial LPP in the absence of scalp disease.^12^

Herein, we presented 14 patients with facial papules who had scalp involvement (FFA or LPP) as well as 5 patients with the isolated facial LPP in the absence of other sites of disease activity and then evaluated the responses to the treatment by isotretinoin.

## 2 CASES' PRESENTATION

This case series study was performed on 19 patients with facial papule who were referred to Alzahra hospital and clinics affiliated with Isfahan University of Medical Sciences in Isfahan province, Iran, during 2018‐2019. Also, it was approved by Ethical Committee of Isfahan University of Medical Science (Ethical code 32917). The patients who met the inclusion criteria were those with the clinical feature of facial papules whose diagnosis was histopathologically proved. To quantify the pretreatment and post‐treatment responses to isotretinoin, we used Global Improvement Scale Assessments (GISA).

In this study, 19 patients were diagnosed with facial LPP who were then enrolled to the study. Except 2 male subjects, all the patients in this series were women (41.17% postmenopausal and 58.82% premenopausal). The patients’ average age was between 32 and 68 years old with mean age ± SD of 49.36 ± 11.61. Moreover, FFA was found in 9 (47.36%) patients who were all women, whereas the classic form of LPP was evident in 5 (29.4%) patients (2 men and 3 women). Five (29.4%) patients, who were all women, were presented only with facial LPP with no other sites involvement.

Concerning the presenting signs and symptoms, 9 (47.36%) patients had been referred with chief complaints of facial skin roughness. Interestingly, some of these patients were misdiagnosed and then underwent laser resurfacing (2 cases) and needle radiofrequency (1 case) due to their lesions. Following these procedures, exacerbation of facial lesions, which suggest Koebner's phenomenon, were observed. In 10 out of 19 cases, facial papule was recognized following the scalp involvement.

Two male and three female patients with LPP of scalp underwent the treatment by systemic drugs (cyclosporine, hydroxychloroquine and or MTX, prednisolone, and phototherapy) for LPP of scalp; however, facial papules respond to these treatments in none of them.

We observed different clinical patterns of facial papules according to age and sex of the patients included. All the patients who were older than 50 years old (postmenopausal women) have subtle clinical expression and had been previously diagnosed with LPP or FFA. On the other hand, facial lesions were better observed in younger patient and many of them presented with such lesions. Moreover, two male patients presented with severe papular eruption over their faces, and in both of them, physical examination revealed LPP of the scalp.

**Figure 1:**
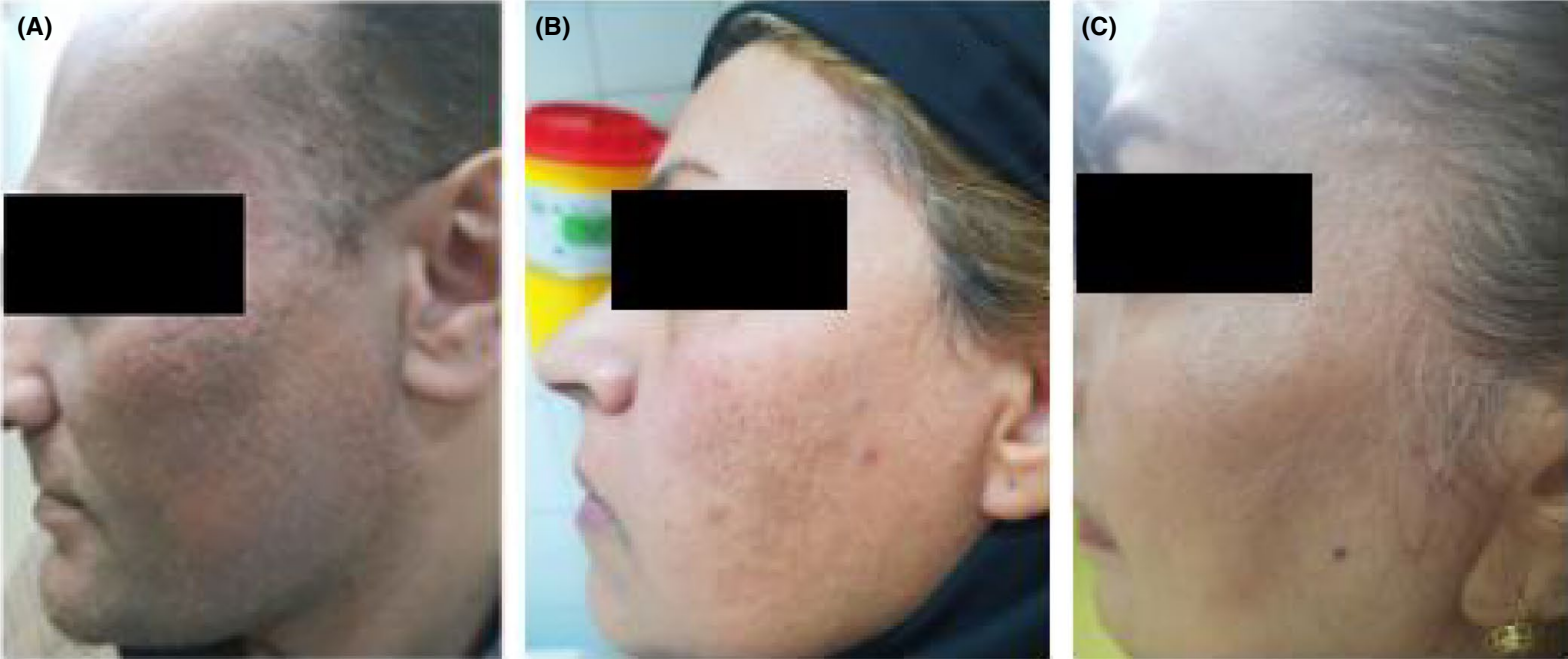
Facial papule in A, a male patient, B, a premenopausal female without frontal fibrosing alopecia (FFA), and C, a postmenopausal female with FFA

The shape of lesions and pattern of their distribution varied between male and female subjects, and also there was a difference between the patients aged under 50 years old and those over 50 years old (Figure 1 and Table 1).

**Table 1:** Clinical presentation in different groups

Male patients	Premenopausal female patients	Postmenopausal female patients
• Sever eruptive skin‐colored papules densely concentrated on temples, forehead and cheeks • More noticeable papules accompanied by atrophic areas • Scalp affected by LPP • No symptoms( itching, irritation) or erythema and scaling	• Skin‐colored papules more concentrated on the cheeks admixed with atrophic areas • Comedo‐like lesions (keratin‐filled dilated infundibula) on the cheeks • Full‐face skin roughness due to tiny skin‐colored papules, especially on the cheeks and around the mouth, • Facial skin may have erythematous or peau d'orange appearance • Facial skin involvement may appear before the scalp alopecia • No symptoms( itching, irritation) or erythema and scaling	• Skin‐colored papular lesions concentrated on the temples, malar prominences, cheeks, and chin area • Peau d'orange or goose bumps appearance of the temples and malar skin • Atrophic glabrous areas devoid of papules along the mandibular inferior margin and preauricular areas. Skin in the affected areas is thin and hypopigmented • Well‐defined hyperpigmented macules pronounced mostly on the medial facial region (cheeks) • No symptoms (itching, irritation) or erythema and scaling • Scalp involvement in the large majority of them (LPP or FFA)

Abbreviations: FFA, Frontal fibrosing alopecia; LPP, Lichen planopilaris.

In 11 of 19 cases (64.7%), eyebrows were affected, partially in 9 patients (47.36%) and totally in 2 patients (10.52%), whereas eyelash loss was presented in 2 patients (10.52%).

**Figure 2:**
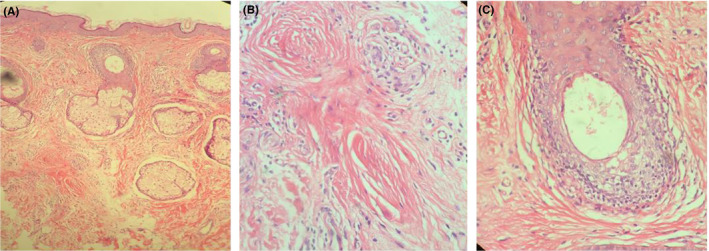
Histologic findings of facial papules

The skin biopsy of facial papules showed lymphocytic infiltration around the vellus hairs accompanied by the vacuolar degeneration of basal epithelium of these hairs as well as the replacement of vellus hairs by fine fibrous tracts (Figure 2).

During the study process, the patients were provided with an adequate explanation on the project and the informed consent was also obtained. Afterward, all the patients were referred to the laboratory for initial blood tests including CBC, BUN, Cr, and LFT, and beta‐HCG for female patients. Also, the patients daily received 20 mg of isotretinoin orally for 6 months. During the treatment course, follow‐up appointments were arranged after 1, 2, 4, and 6 months of treatment. Comparison of the changes in lesions and the 4‐point grading scale score (Table 2) from base line was performed, based on photography.

**Table 2:** Response to treatment in patients based on dermatologist's opinion

Response to treatment		Frequency	Percent
	Non‐response	1	5.3
	Mild to moderate	5	26.3
	Good	8	42.1
	Very good	5	26.3

**Figure 3:**
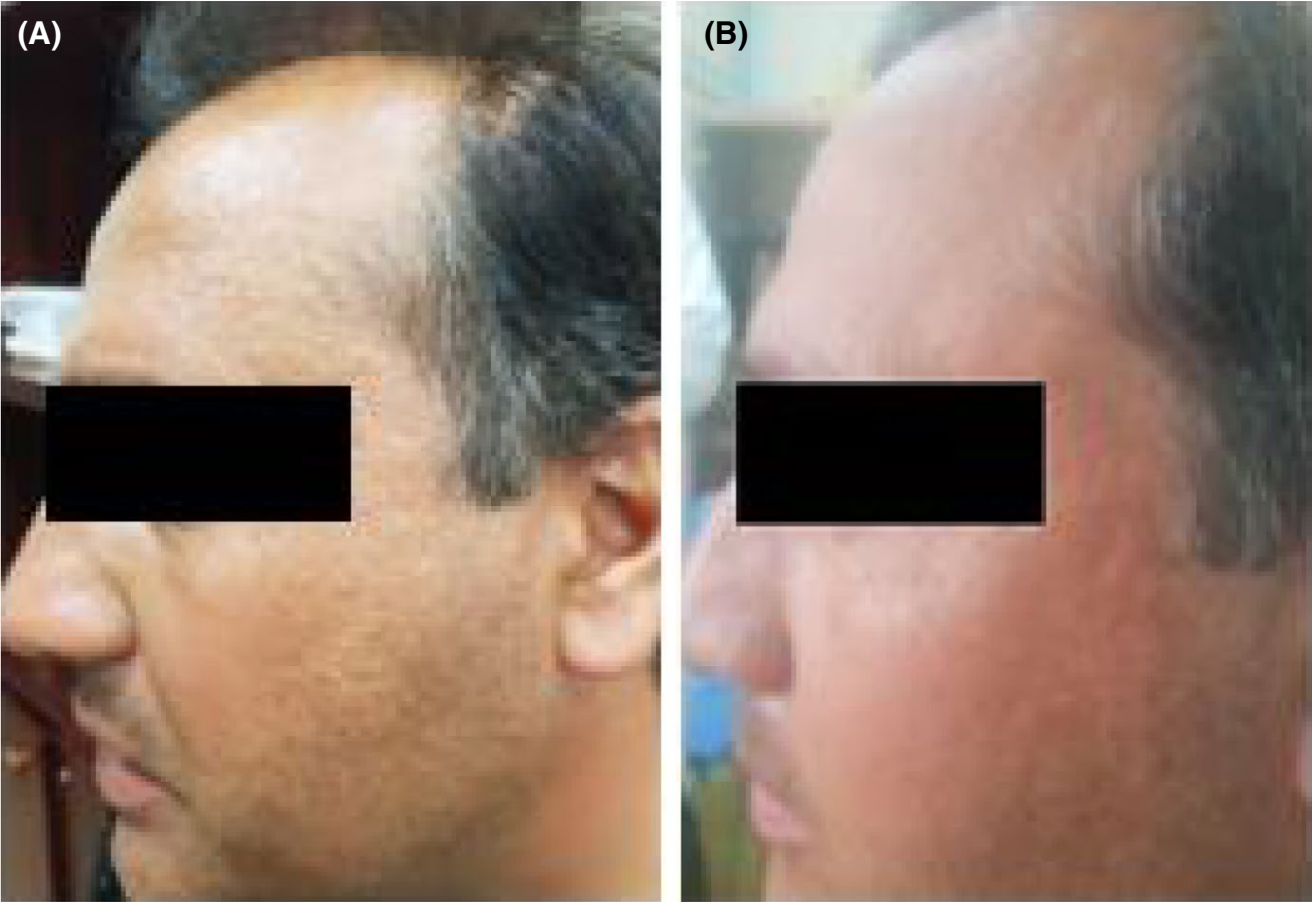
A, Before and B, after treatment with isotretinoin in a male patient

After 6 months of treatment, before and after intervention's photographs were scored by a blind dermatologist. Notably, the response to the treatment was dramatic in 2 men, which was significantly varied in female patients; and in a way, the lesions have significantly reduced after 6 months of treatment. 10 patients (58.8%) were satisfied with the treatment and 3 cases (17%) had satisfactory results. Finally, the papular lesions have clearly reduced, and the skin was smoother, especially in male patients (Figure 3); however, this treatment did not affect the FFA and LPP of scalp.

Improvement with oral isotretinoin was apparent in all the patients within this 6‐month period. Many papular lesions rapidly disappeared and a smooth skin remained. At the final visit, 63.15% of the patients stated that they were satisfied with the results of the treatment. Interestingly, scalp disease was not affected by such treatment.

The scoring system used to evaluate the treatment results by a dermatologist was as follows: 0 = no response, 1 = mild to moderate response, 2 = good response, and 4 = a very good response.

The responses to the treatment in the patients were evaluated by a blinded dermatologist based on her opinion. In this regard, 42.1% of the patients had a good response and 26.3% of them had a very good response (Table. 2).

## 3 DISCUSSION AND CONCLUSION

Lichen planopilaris is a scaring alopecia presented as patchy or diffuse hair loss perifollicular erythema, perifollicular scaling, and follicular keratosis. Accordingly, it can be subdivided into 3 variants, including classic LPP FFA and Graham‐Little‐Piccardi‐Lasseur syndrome.^1^

Frontal fibrosing alopecia was firstly described as a progressive loss of frontotemporal hairlines in postmenopausal women by Kossard in 1994.^13^ Although premenopausal women or even men might be affected, we found a huge number of premenopausal women affected by FFA (36.8%). In our series, we had 2 men (10.5%) who presented more severe facial papules compared to women. Recently, the involvement of facial vellus hairs presenting as peculiar facial papules has been reported to be associated with FFA. On the other hand, the disease might extend beyond the frontotemporal hair line and also affect peripheral body hair, which eventually lead to loss of eyebrows and eyelashes. Thus, FFA has been recognized as a generalized rather than localized process.^14^

Frontal fibrosing alopecia may be associated with clinical or serologic evidence of autoimmunity, for example, hypothyroidism. Moreover, in concordance with previous reports, we found a high prevalence of hypothyroidism. Among the patients in this study, there were 3 cases (15.7%) of hypothyroidism and 3 cases (15.7%) of vitamin D deficiency.

Facial vellus hair involvement presented as noninflammatory facial papules has been described to be associated with FFA.^8^ The clinical picture includes follicular micropapules that are randomly distributed over the facial skin, which are readily more visible over temporal and cheek regions. At the scalp biopsy, an inflammatory lymphocytic infiltrate around the upper portion of hair follicle can be observed with the common findings of perifollicular lamellar fibrosis and fibrosis of follicular tract ^15^; therefore, it can be considered as a variant of LPP.

In our experience, it was shown that these skin changes are more prominent in male patients due to terminal nature of facial hair follicles. On the other hand, elderly patients may have subtle clinical expression because of coexisting of wrinkles and solar elastosis. Also, premenopausal women present with more evident papules even before scalp or eyebrows alopecia.

In parallel with our observation, Maele et al have described facial LPP in the absence of FFA in the premenopausal women.^12^ In the current study, we found facial LPP in 9 women (47.3% of our case) in the absence of scalp involvement, and out of them, 7 cases (36.8%) were premenopausal. Because of these findings, this question comes to mind that whether these facial papules are in the clinical spectrum of FFA or a variant of LPP that can occur as a single presentation without involving any other body sites. In this regard, more studies with longer follow‐ups are needed to recognize the nature of these lesions.

In some studies, histological evaluation of these papules revealed the elastic fiber involvement accompanied with the preserved large sebaceous glands.^16^

Retinoids including isotretinoin are known to perform several actions such as anti‐inflammatory effects and induction of apoptosis in sebaceous glands..^17^ Accordingly, there are some reports of successful treatment of FFA with topical retinoids.^18^ Also, there is a report of successful treatment of the facial papules in FFA with oral administration of isotretinoin.^16^ In addition, in our practice we have never observed FFA or LPP patients who have improved their facial lesions by using other treatments (eg, hydroxychloroquine, cyclosporine, methotrexate, and prednisolone).

These observations prompted us to conduct this study to evaluate the therapeutic effects of oral isotretinoin as a first‐line treatment of facial LPP. The obtained results are promising, which show that the facial papules dramatically disappeared (42.1% good and 26.3% very good response). It seems valuable to consider the use of oral isotretinoin for the treatment of facial papules with LPP nature. However, more studies with greater numbers of patients are still necessary to better evaluate the efficacy of oral isotretinoin optimum dosage, treatment duration, and comparison between topical and systemic retinoids as well as a long‐term follow‐up of the patients and determining the incidence of recurrence after stopping the treatment. On the other hand, isotretinoin‐induced sebaceous gland atrophy is not permanent, so a long‐term follow‐up is necessary to confirm our observations.

## CONFLICT OF INTEREST

None declared.

## AUTHOR CONTRIBUTIONS

Farahnaz Fatemi involved in study design and data collection. Fatemeh Mohaghegh involved in study design and paper wring. Farzaneh Danesh involved in study design, data collection, involved in data analysis. Mina saber involved in paper editing and data analysis. Parvin Rajab involved in study design.
